# Antibacterial Nanostructured Coatings

**DOI:** 10.3390/nano13232982

**Published:** 2023-11-21

**Authors:** Loredana Tammaro

**Affiliations:** ENEA—Italian National Agency for New Technologies, Energy and Sustainable Economic Development, Nanomaterials and Devices Laboratory (SSPT-PROMAS-NANO), Piazzale E. Fermi, 1, 80055 Portici, NA, Italy; loredana.tammaro@enea.it

## 1. Introduction

Nanomaterials, which by definition must have at least one of their constituents at the nanoscale, can display unique optical, magnetic, electrical, mechanical, and other properties [1,2]. Consequently, nanomaterials have led to the successful development of nanostructured coatings [3,4].

Nanostructured coatings are particularly suited to protecting surfaces and are therefore gaining interest in different fields, such as medicine, food manufacturing, textiles, marine coatings, water treatment, electronics, construction, automotive vehicles, and energy [5,6,7]. 

Furthermore, the incorporation of an antimicrobial agent, capable of killing or at least inhibiting the growth of pathogenic microorganisms, adds the advantage of active species to coatings at the nanometer level. Commonly used antimicrobial agents are metal ions, polymers, antimicrobial peptides, quaternary ammonium compounds, naturally derived antimicrobials (i.e., essential oil), etc. [8,9].

In this framework, the journal *Nanomaterials* has dedicated a successful Special Issue to the topic “Antibacterial Nanostructured Coatings”.

The Special Issue “Antibacterial Nanostructured Coatings” collects high-quality articles in this research area. The current Special Issue compiles six papers. Several procedures have been exploited for the preparation of antibacterial coatings with the aim of protecting surfaces from microbial contamination. 

The original articles include studies on the potential applications of the investigated antibacterial nanostructures and examine different uses, such as devices in the healthcare sector [10], suture materials [11], food packaging films [12], water treatment membranes [13], titanium-based alloys for medical implants [14], and tissue engineering scaffolds [15]. 

Laube et al. compared ultrathin polymeric coatings based on vinyl benzyl monomeric units bearing quaternary ammonium moieties prepared using two different methods with regard to their antimicrobial activity on titanium surfaces. Their coatings showed good substrate adhesion, cytocompatibility under in vitro conditions, and good antimicrobial activity depending on the length of the alkyl chain of the quaternary ammonium compounds. Possible applications include equipment of metallic devices used in healthcare and medical implants [10]. Basov et al. found that, after the application of the freeze/thaw method, the antibacterial activity of suture materials with Ag nanoparticles (AgNPs) was significantly higher than before their treatment with cyclic freezing. The application of the developed technique led to the overwhelming predominance of nanoparticles with 1 to 15 nm diameter, which can be used in medical practice for the additional processing of fibers with AgNPs and for obtaining suture materials containing mainly small-diameter nanoparticles [11]. 

With a different application purpose, Viscusi et al. evaluated the activity of bio-coatings based on pectin loaded with antimicrobial grapefruit seed oil (GO) encapsulated into halloysite nanotubes for the preservation of fresh fruits. The release of linoleic acid, the main component of GO, was correlated with the amount of hybrid filler, demonstrating the possibility of tailoring the release kinetics of active molecules and applying the prepared formulations in the food packaging field [12].

In this Special Issue, Fiaschini et al. proposed electrospun polysulfone (PSU) membranes modified with sputtered Ag nanocoatings to prevent/reduce fouling. Despite the very low amount of Ag deposited on the polymer fibers, this functionalization was able to strongly reduce the microbial growth on the developed electrospun membranes, making them promising for application in ultra-filtration and reverse osmosis in water treatment [13]. Lallukka et al. developed a biocompatible and antibacterial surface of Ti6Al4V-ELI alloy by functionalization with peptide nisin. The antibacterial performance of the adsorbed nisin coating assessed against the Staphylococcus aureus strain showed promising anti-microfouling activity, and a possible effect on biofilm maturation was preliminarily obtained [14]. Mesoporous silica-based bioactive glass (MBG) sub-micrometric particles, as carriers for *Melaleuca Armillaris* essential oil and gentamicin, for the prevention of intrahospital infections were reported by Ballarre et al. [15]. They demonstrated the high bioactivity of the MBG particles as apatite precursors for bone repair and an antibacterial behavior against Gram-negative bacteria. Such systems are therefore interesting building blocks for applications such as coatings and tissue engineering scaffolds.

In summary, I am confident that this Special Issue will contribute to the research interest in the field, providing our readership with recent studies that outline the importance of developing nanostructured coatings with antibacterial activity for application in several fields.

Overall, I am grateful to all the authors for their meaningful contributions to the present Special Issue, and I hope that scholars will pay attention to these published works for forthcoming studies and new applications of antibacterial nanostructured coatings.

## Data Availability

No new data were created or analyzed in this study. Data sharing is not applicable to this article.
